# Glasgow Coma Scale Score at Admission in Traumatic Brain Injury Patients: A Multicenter Observational Analysis

**DOI:** 10.3390/jcm14155195

**Published:** 2025-07-22

**Authors:** Iulia-Maria Vadan, Diana Grad, Stefan Strilciuc, Emanuel Stefanescu, Olivia Verisezan Rosu, Marcin Michalak, Alina Vasilica Blesneag, Dafin Muresanu

**Affiliations:** 1 Department of Neurosciences, “Iuliu Hațieganu” University of Medicine and Pharmacy, 400012 Cluj-Napoca, Romania; stefan.strilciuc@brainscience.ro (S.S.); alinablesneag@yahoo.com (A.V.B.); dafinm@ssnn.ro (D.M.); 2 RoNeuro Institute for Neurological Research and Diagnostic, 400012 Cluj-Napoca, Romania; diana.grad@brainscience.ro (D.G.); stefanescu.emanuel@yahoo.com (E.S.); dr.olivia.rosu@gmail.com (O.V.R.); 3 Department of Genomics, MEDFUTURE Institute for Biomedical Research, “Iuliu Hațieganu” University of Medicine and Pharmacy, 400012 Cluj-Napoca, Romania; 4 Department of Stroke and Neurology, Saint Vincent’s Hospital, 81-348 Gdynia, Poland; michalak83@gmail.com; 5 Neurology Clinic, Cluj County Emergency Clinical Hospital, 400347 Cluj-Napoca, Romania

**Keywords:** traumatic brain injury, Glasgow Coma Scale, clinical outcome, socio-demographic outcome, hospital outcomes, Eastern Europe, prognostic factors, socioeconomic factors, retrospective study

## Abstract

**Introduction:** Traumatic brain injury (TBI) is a leading cause of morbidity worldwide, with the Glasgow Coma Scale (GCS) serving as a tool to measure injury severity. This study aimed to investigate the relationship between GCS admission scores and various socio-demographic, clinical, injury-related, and hospital-related variables in patients with TBI across two tertiary care centers in Eastern Europe, a region that remains underrepresented in the literature. **Methods:** A retrospective observational study was conducted using data from 119 TBI patients admitted between March 2020 and June 2023 at Cluj County Emergency Hospital (Romania) and Saint Vincent Hospital (Poland). GCS scores were analyzed as both categorical and continuous variables. Statistical analyses included Wilcoxon and Kruskal–Wallis tests for group comparisons and Spearman correlations for continuous variables. **Results:** Most patients included suffered a mild TBI (GCS score between 13 and 15). There were significant associations between GCS scores and post-traumatic amnesia (*p* < 0.05), discharge status (*p* < 0.01), discharge destination (*p* < 0.01), and education level (*p* < 0.01). GCS scores at admission were linked to survival, absence of post-traumatic amnesia, higher education levels, and home discharge. No significant differences observed across sex, residence, employment status, injury type, cause, or mechanism of injury. A weak but significant negative correlation was observed between GCS and length of hospital stay (rho = −0.229, *p* > 0.05), while age showed a non-significant correlation. **Conclusions:** The GCS score at admission is significantly associated with various clinical and socio-demographic outcomes in TBI patients, supporting the utility of the GCS score as a prognostic tool. The predominance of mild cases and the absence of radiological data, such as cerebral contusions or epidural or subdural hematomas, limit the generalizability of the findings. Further studies with larger samples and comprehensive imaging data are necessary to validate these findings.

## 1. Introduction

Traumatic brain injury (TBI) represents one of the most frequent and critical neurologic pathologies, with more than 70 million people globally suffering from a TBI yearly, and in the European Union, there are approximately 1.5 million people that need hospitalization for TBI every year [1,2,3], posing a growing burden on healthcare systems, especially in aging populations [4]. Lower- and middle-income countries present the highest severity and mortality due to the injuries suffered [5]. It is estimated that half of the world population will suffer from a TBI in their lifetime [6]. Approximately 80% of all TBIs diagnosed in the United States are considered to be mild, and mild-to-moderate ones constitute almost 90% of all TBIs [5,7].

TBI remains an important neuropathology, and it is classified depending on several factors, the most important, especially in the acute state, being the Glasgow Coma Scale (GCS) [6].

The Glasgow Coma Scale includes three variables: eye-opening, verbal, and motor, widely used for assessing the neurologic status of patients after TBI. It represents one of the essential tools in triage, determining the appropriate level of care, and assessing TBI severity, categorizing the entity into mild (GCS between 13 and 15), moderate (GCS between 9 and 12), and severe (GCS ≤ 8) [1,8,9,10,11]. Multiple studies have attempted to determine which out of the three evaluated components is the most relevant. While most studies have explored the predictive value of the motor component, others have revealed the prognostic importance of the eye-opening and verbal components [12]. It was also shown that determining the GCS score early after a TBI was correlated with subsequent clinical outcomes and in-hospital mortality [13,14,15].

Beyond its clinical application, the GCS score is influenced by a diverse range of factors. For example, lower education and rural residence have been associated with more severe injuries, and thus lower GCS scores [16,17]. Radiological findings are important in determining the severity of a TBI and help predict the expected outcome. For example, imaging features like cerebral contusions and epidural or subdural hematomas are correlated with lower GCS scores and, as a result, increased mortality [18,19,20].

Despite the wide use of GCS, data from Eastern European countries remain underrepresented in the literature. This study aimed to examine the correlations and group comparisons between GCS scores at admission and various socio-demographic, clinical, injury-related, and hospital-related variables in TBI patients from two tertiary care centers in Eastern Europe: Cluj County Emergency Hospital in Romania and Saint Vincent’s Hospital in Gdynia, Poland.

## 2. Methods

### 2.1. Study Design

We conducted a retrospective, observational study using data from consecutive TBI patients aged over 18 who were admitted between March 2020 and June 2023 to two hospitals—one from Romania (Cluj County Emergency Hospital) and one in Poland (Saint Vincent Hospital in Gdynia).

### 2.2. Data

The database contained a total of 180 TBI patients; however, only 138 had data for the outcome variable—GCS. We conducted a complete case analysis and excluded patients with missing data for at least one of the other included variables, resulting in a final sample of 119 TBI patients.

We analyzed different types of variables, such as socio-demographics (sex, residence—binary variable; age—continuous variable; education, employment, marital status—nominal variables); clinical data (GCS admission score—continuous; existence of post-traumatic amnesia—binary variable; and Modified Marshall score—nominal variable); injury-related variables (place of injury, cause of injury, mechanism of injury, type of injury—nominal variables); and hospital-related variables (discharge status—binary variable; discharge location—nominal variable; and hospital length of stay—continuous variable). Sex includes females and males. Education has 6 categories (primary school (1–4 years), secondary school (5–8 years), high school (9–12 years), university (13+ years), no formal education, and unknown). Employment has 5 categories (employed, unemployed, retired, unknown, and other). Marital status has 6 categories (married, single, separated, living together, widowed, and unknown). Residence is categorized as either rural or urban. Post-traumatic amnesia has two categories: yes and no. The place of injury has 5 categories (home, work, other, street, and sports facility). The cause of injury has 4 categories (aggression, fall, road traffic accident, and other). Mechanism of injury has 8 categories (fall from height > 1 m (3ft), ground-level fall, other non-penetrating TBI, direct impact: blow to the head, acceleration/deceleration, direct impact: head against an object, and missing data). The type of injury has three categories: closed, penetrating, and crush. Discharge status is categorized into two categories: alive and dead. Discharged to has 4 categories: home, another hospital, rehabilitation, and others. The Modified Marshall score had 5 categories: I, II, III, IV, and V.

The primary variable of interest is the GCS score (severe—score of 8 or less; moderate—between 9 and 12; mild—between 13 and 15). Although it was collected as a continuous variable, we also use it as a categorical variable to characterize the study sample when reporting descriptive statistics; however, when conducting statistical tests, it is used as a continuous variable.

### 2.3. Statistical Analysis

We present counts and percentages for the categorical (binary and nominal) variables stratified by GCS categories. For continuous variables, we present both the mean and median.

We conducted Shapiro–Wilk tests for GCS scores, age, and hospital length of stay. The results showed that the distributions of GCS scores at admission (W = 0.47, *p*-value < 0.01), age (W = 0.93, *p*-value < 0.01), and hospital length of stay (W = 0.73, *p*-value < 0.01) departed significantly from normality. Thus, we used non-parametric tests such as the Wilcoxon rank-sum test, the Kruskal–Wallis test, and the Spearman correlation. In addition, as our sample is predominantly formed of mild patients, we also conducted Shapiro–Wilk tests for this subgroup. The results showed, as in the case of the entire sample, that the distributions of GCS scores at admission (W = 0.49, *p*-value < 0.01), hospital length of stay (W = 0.70, *p*-value < 0.01), and age (W = 0.93, *p*-value < 0.05) departed significantly from normality. In addition, we inspected visually the variables, which suggests that the data is not normally distributed.

As with the entire sample, we used the same non-parametric tests: the Wilcoxon rank-sum test, the Kruskal–Wallis test, and the Spearman correlation. The statistically significant Kruskal–Wallis test results were afterwards subjected to post hoc pairwise comparisons with the Bonferroni correction to identify groups that exhibited statistically significant differences.

To compare GCS scores among groups, we used Wilcoxon test for categorical variables that had two subgroups (sex, residence, post-traumatic amnesia, and discharge status) and Kruskal–Wallis for variables with more than two subgroups (education, marital status, employment, Modified Marshall score, type of injury, place of injury, cause of injury, mechanism of injury, and discharge location).

In addition, we used Spearman correlation for GCS scores and age, as well as for GCS scores and length of stay (continuous variables).

All analyses were conducted using R v4.3.3. The significance value was set at 0.05.

## 3. Results

### 3.1. Descriptive Statistics

Our sample consisted of 119 patients. When stratified by GCS severity categories, most patients were classified as mild (n = 106), regardless of the subgroups of each of the variables presented in Table 1. As for the total sample, most patients were male (n = 77), university-educated (13+ years) (n = 59), retired (n = 71), married (n = 54), and resided in urban areas (n = 87). Most TBI patients reported having no post-traumatic amnesia (n = 86), were injured at home (n = 76), had a fall as a cause of injury (n = 91), and listed ground-level fall as a mechanism of injury (n = 72). In 114 cases, the type of injury was closed. Most patients were discharged alive (n = 107) and were discharged home (n = 104).

**Figure 1:**
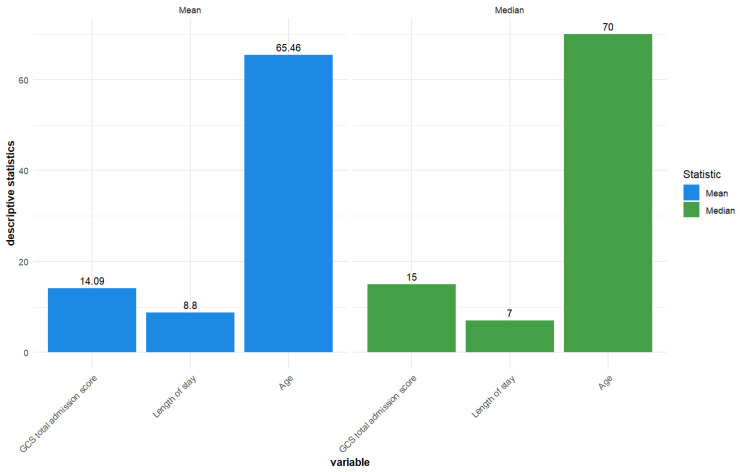
Descriptive statistics—Mean and median for GCS total admission score, length of stay, and age for the included TBI patients (n = 119).

The mean length of stay was 8.8 days, average GCS total admission score was 14.09, and mean age was 65.46 years. The median length of stay was 7, the median GCS total admission score was 15, and the median age was 70 years (Figure 1).

**Figure 2:**
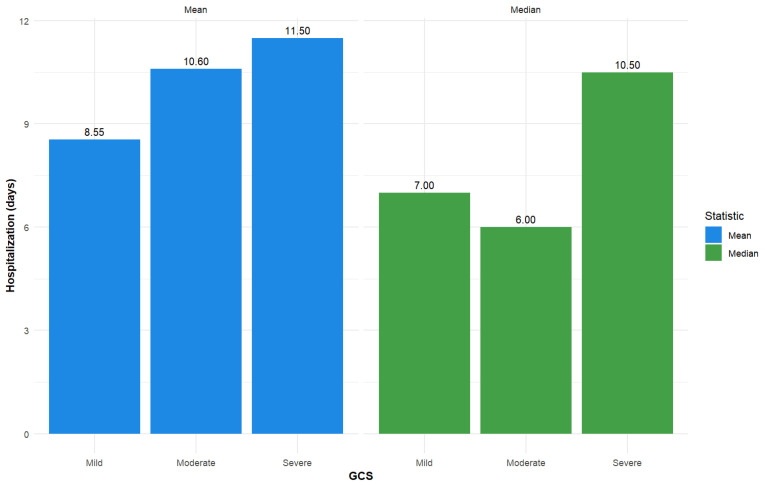
Descriptive statistics—Mean and median for inpatient hospitalization (days) for GCS total admission score (used as a categorical variable; severe (GCS ≤ 8), moderate (GCS 9–12), and mild (GCS 13–15)).

**Figure 3:**
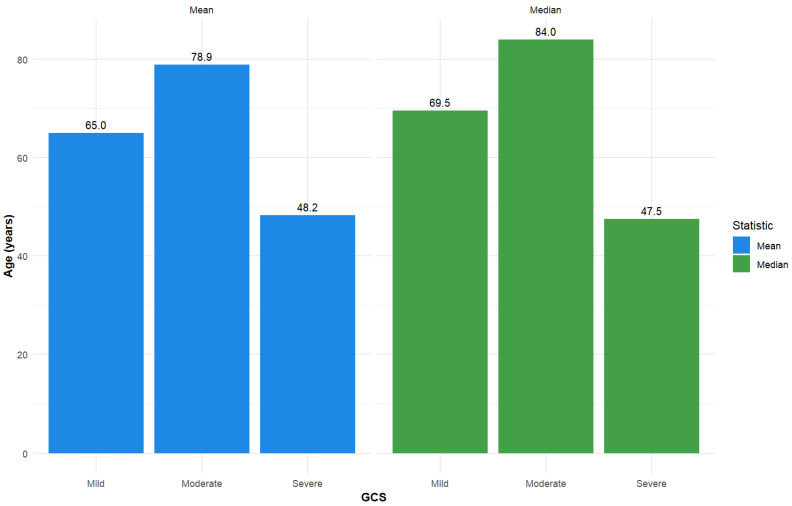
Descriptive statistics—Mean and median for age, stratified by GCS total admission score (used as a categorical variable: severe (GCS ≤ 8), moderate (GCS 9–12), and mild (GCS 13–15)).

Additional descriptive statistics for length of stay and age are presented in Figure 2 and Figure 3.

### 3.2. Wilcoxon Results

Based on the Wilcoxon analyses, we found no statistically significant differences in the GCS total admission score for categories of sex (W = 1442.5, *p* > 0.05, with the estimated difference in location being of −0.00002; 95% CI: −0.00003 and 0.00006) and residence (W = 1570.5, *p* > 0.05, with the estimated difference in location being of 0.00005; 95% CI: −0.00001 and 0.00002). However, we found statistically significant differences for the categories post-traumatic amnesia (W = 1732.5, *p* < 0.05, with the estimated difference in location being of 0.00001; 95% CI: 0.00002 and 0.00003) and discharge status (W = 1011, *p* < 0.01, with the estimated difference in location being of 1.99999; 95% CI: 0.99995 and 3.00006).

For patients with mild TBI, statistically significant differences were observed in the GCS total admission scores for post-traumatic amnesia (W = 1322, *p* < 0.05), with an estimated difference in location being of 0.00005 (95% CI: −0.00002 and 0.00002). Sex: W = 1209, *p* > 0.05; estimated difference in location: 0.00000; 95% CI: −0.00006 and 0.00007, residence: W = 1197.5, *p* > 0.05; estimated difference in location: 0.00004; 95%: −0.00006 and 0.00004, and discharge status: W = 397, *p* > 0.05; estimated difference in location: 0.00005; 95% CI: −0.00005 and 0.99994.

### 3.3. Kruskal–Wallis

Based on the Kruskal–Wallis analyses, we found statistically significant differences in GCS total admission scores for the subgroups discharge destinations (χ^2^ = 23.80, *p* < 0.01), education levels (χ^2^ = 24.16, *p* < 0.01), marital status (χ^2^ = 13.13, *p* < 0.05), and Modified Marshall Score (χ^2^ = 10.86, *p* < 0.05). However, there were no statistically significant differences in GCS total admission scores for the subgroups type of injury (χ^2^ = 5.10, *p* > 0.05), place of injury (χ^2^ = 6.93, *p* > 0.05), cause of injury (χ^2^ = 3.50, *p* > 0.05), mechanism of injury (χ^2^ = 7.32, *p* > 0.05), or employment status (χ^2^ = 1.36, *p* > 0.05). The results of the post hoc analysis (combined with Bonferroni corrections) for the statistically significant results showed that there were differences in the GCS total admission score among TBI patients having a lower level of education—secondary school—and those with higher education—university (*p* < 0.05), as well as among TBI patients whose level of education was unknown and those whose last level of reported education was the university level (*p* < 0.01). For the remaining pairwise comparisons, no other statistically significant differences were found.

Regarding discharge location, there were differences in the GCS total admission score of TBI patients discharged to another hospital and home (*p* < 0.05) as well as between those discharged home and to other destinations (*p* < 0.01). As for the remaining pairwise comparisons, no statistically significant differences were found.

No statistically significant differences were found in any of the subgroups of the Modified Marshall score and marital status.

For patients with mild TBI, we found statistically significant differences in GCS total admission score among the subgroups marital status (χ^2^ = 11.28, *p*-value < 0.05), while there were no statistically significant differences in the subgroups education (χ^2^ = 9.10, *p* > 0.05), employment (χ^2^ = 2.52, *p* > 0.05), place of injury (χ^2^ = 5.50, *p* > 0.05), cause of injury (χ^2^ = 2.92, *p* > 0.05), mechanism of injury (χ^2^ = 7.49, *p* > 0.05), type of injury(χ^2^ = 0.46, *p* > 0.05), discharge to (χ^2^ = 7.63, *p* > 0.05), and Modified Marshall Score (χ^2^ = 7.00, *p* > 0.05). Following the post hoc analysis (combined with Bonferroni corrections) for the statistically significant results regarding marital status, no further statistically significant results were found.

### 3.4. Spearman Correlations

Based on the results for the Spearman correlations, there was a statistically non-significant negative correlation for GCS and age (rho = −0.175, 95% CI: −0.344, and 0.004, *p* > 0.05), while for GCS and hospital length of stay, there was a statistically significant stronger negative correlation (rho = −0.229, 95% CI: −0.392, and −0.051, *p* > 0.05).

The results were similar for patients with mild TBI, as there was a statistically non-significant negative correlation for GCS and age (rho = −0.159, 95% CI: −0.340, and 0.032, *p* > 0.05), while for GCS and hospital length of stay, there was a statistically significant stronger negative correlation (rho = −0.313, 95% CI: −0.475, and −0.130; *p* < 0.05).

## 4. Discussion

The primary objective of this study was to examine group comparisons and correlations between the GCS admission score and various clinical, socio-demographic, injury-related, and hospital-related variables in a cohort of TBI patients admitted to two European hospitals, one located in Romania and the other in Poland. The main findings revealed several crucial associations that can contribute to understanding TBI severity and its relationships. Several articles have investigated the impact of the GCS score at admission on the outcome of patients with TBI, and the results have shown a significant influence. Some studies have demonstrated that a score ≤ 8 points is an essential predictor for the mortality of patients [21,22].

The majority of patients from the 119 group that we monitored presented with mild TBI, a finding that was consistent across nearly all demographic and injury-related divisions. Patients had a mean age of 65.46 years, and the predominant cause of injury was falls. This finding is in contrast with other studies from the literature, where elderly patients (defined by the United Nations and the World Health Organization as adults aged ≥ 60 years, while a more generally used definition is ≥ 65 years) with a predominance of injuries caused by falls commonly suffer more severe TBIs [23,24,25]. The mean age in the case of severe TBI was 48.2. Other studies have also shown a tendency for more severe TBIs in young persons [26,27], but lower chances of mortality and higher chances of recovery compared to the older patients [28,29].

Leitgeb et al. demonstrated in their research that the GCS score at ICU discharge was a reliable predictor of the 1-year outcome for patients [16]. Our results showed that post-traumatic amnesia and discharge status were significantly associated with GCS admission scores. The patients who did not experience post-traumatic amnesia had significantly higher GCS scores, which suggests less severe neurological injuries. Moreover, patients who survived hospitalization had higher GCS scores at admission than those who did not, confirming the predictive value of GCS as a prognostic tool in TBI, as previously established in the literature [30,31].

Discharge destination and education level also showed significant differences in GCS scores at the time of patient admission. Previous research has shown that a higher level of education increases the likelihood of a patient being admitted to the intensive care unit (ICU) compared to home discharge, but does not increase the likelihood of admission to a floor setting [32]. Also, a lower education level was linked to higher rates of cognitive impairment after a TBI [17]. In our research, patients discharged home had higher GCS scores. In comparison, those transferred to other facilities had lower scores, indicating that patients with less severe injuries were more likely to be discharged directly home. There were group differences in our sample for the GCS admission score, with patients who had a higher education presenting with higher GCS scores compared to those with lower or unknown levels of education. This may suggest an indirect relationship between socioeconomic status and injury severity or recovery potential, a relationship previously documented in TBI outcomes research [33,34].

Other demographic variables, including sex, residence, and employment status, did not show significant associations with GCS score. In contrast, prior research has shown differences between genders in the severity of the TBI based on the GCS scores [35,36]. Furthermore, it was suggested that residence could influence the GCS score at admission if patients do not have access to healthcare facilities in their region and prehospital care varies between regions [16]. Similarly, variables related to the type, cause, and mechanism of injury were not significantly associated with GCS scores. A possible cause for these results is that our cohort has a predominance of fall-related injuries across all GCS categories, which reduced the variability and statistical contrast between subgroups. In addition, these factors alone might not be sufficient predictors of the patient’s neurological status at presentation.

The Modified Marshall Score, commonly used to evaluate the extent of structural damage to the brain, and marital status showed significant Kruskal–Wallis results. A post hoc analysis did not reveal any statistically significant pairwise differences compared to other studies, which showed a direct link between the GCS score and the Modified Marshall Score [37]. This may be due to small sample sizes for subgroups (underpowering the post hoc pairwise analyses)as well as the aim of the Bonferroni correction, which aims to reduce false positives (Type 1 errors) [38]. This emphasizes the need for further studies with a higher number of participants. Additionally, the absence of detailed imaging findings, such as the presence of cerebral contusions and epidural or subdural hematomas, limits the ability to draw stronger pathophysiological connections, which represents a notable limitation of this study.

Spearman correlation analyses revealed a small, non-significant negative association between age and GCS score, aligning with the evidence that older patients may have poorer outcomes. In contrast, in the literature, many cases show the contrary; young patients tend to have more severe injuries and, as a result, a lower GCS score [26,27,39,40]. We also found a statistically significant negative correlation between GCS and length of hospital stay, indicating that lower GCS scores at admission are associated with more extended hospitalizations. This translates to the fact that the more severe the TBI, the longer the treatment and recovery periods it requires. This result is consistent with other findings in the literature [16,22,41].

The current study does not specify if patients suffered diverse traumatic pathologies such as subdural or epidural hematomas and cerebral contusions, known for their influence on mortality and short and long-term outcomes. These injuries usually present with lower GCS scores and need urgent treatment to obtain the best results. Cerebral contusions are known to have a poorer outcome when they present with a low GCS score, occur in older age, and have a larger initial contusion volume. Moreover, the need for craniotomy and the duration of the ICU stay also influence the outcome [18].

In the case of hematomas, other factors besides the GCS score must be taken into consideration, such as age, pupil abnormalities, hematoma type and thickness, the presence of coagulopathy, glycemia, antithrombotic use, systolic blood pressure, respiratory rate, and blood saturation. The literature indicates that patients with epidural hematomas have a higher survival rate, more favorable outcomes, and lower in-hospital mortality rates [19,20].

Following Bonferroni correction, there were few statistically significant differences between particular subgroups, even though certain variables such as education level, marital status, discharge destination, and Modified Marshall Score, demonstrated statistically significant global associations with GCS scores based on Kruskal–Wallis tests. Although patients with university-level education had significantly higher GCS scores compared to those with secondary education and unknown education status, no significant differences were observed across other education categories. Similarly, although discharge destination and marital status showed significant overall correlations with GCS scores, individual pairwise comparisons failed to reach significance, perhaps due to the small sample sizes in some subgroups. These results suggest that although general patterns exist, they may not accurately represent significant variations within particular categories and should be interpreted cautiously in light of the sample’s composition and size.

## 5. Strengths and Limitations of the Study

This study has several strengths. Firstly, the current study targets a critical global health issue (TBI) and describes its burden, especially in an aging population. Secondly, it is a multicenter study with a comprehensive dataset, which includes a wide variety of socio-demographic, clinical, injury-related, and hospital-related variables. Moreover, it is among the few comparative studies that focus on patient cohorts from two Eastern European countries, Poland and Romania, offering a useful presentation of TBI and highlighting the outcomes that are more frequent in these healthcare systems.

Clinically, this study supports the usefulness of the GCS score as a predictor of hospitalization outcomes, including mortality and length of stay, as well as a triage tool. Moreover, it emphasizes the importance of incorporating cognitive and social contexts in early assessment and discharge planning.

The statistical analysis employed non-parametric tests appropriate for the data distribution and sample size. Lastly, this study also reports a sub-analysis for patients with mild TBI.

This study has several limitations. First, the sample size is relatively small and was not based on an a priori power analysis (which reduces the statistical power of the results). In addition, considering that the results of the Shapiro–Wilk tests indicated that the data for the outcome of interest—GCS admission scores—deviated from normality, we could only use non-parametric tests. Secondly, our sample is primarily composed of patients with mild TBI. This limits the generalizability of the findings to more severe cases. Furthermore, our study focuses only on the adult population, excluding pediatric cases. Moreover, this study does not provide information on whether patients suffered more complicated injuries, such as cerebral contusions or epidural or subdural hematomas. These are clinically significant factors that can affect the neurological outcome both in the short and long term following a TBI. Therefore, the absence of such data makes it more difficult to accurately describe the degree of brain damage in the study population and could result in variability that was not taken into consideration in the analysis. Therefore, results should be interpreted cautiously, especially when evaluating their relevance to patients with more severe or complex intracranial pathologies. Lastly, as our analysis is based on complete cases, we acknowledge that selection bias may occur. Therefore, considering that this study was an exploratory, retrospective exercise, we suggest that future studies should aim for a larger sample size and a more diverse population in terms of age and TBI severity. Additionally, data collection should be based on a priori power analysis and, if possible, data should be collected at multiple time points and in multiple settings (but only in inpatient hospital settings), in order to capture different types of declines or improvements in TBI patients.

## 6. Conclusions

In conclusion, our study highlights the utility of the GCS score as a crucial tool for assessing clinical severity and predicting potential outcomes and pathological courses, including discharge destination and length of stay. The associations with education level and post-traumatic amnesia highlight the importance of taking into consideration socio-demographic and cognitive factors in TBI assessment and management. These findings are especially relevant for Eastern European healthcare systems, where limited data exists. Understanding how the GCS score at admission can influence the outcome of patients can guide better resource allocation, emergency protocols, and post-acute planning.

More studies with larger samples are needed to fully comprehend its effect and better understand its repercussions on the functional outcome of patients.

## Figures and Tables

**Table 1:** Descriptive statistics for 119 TBI patients.

Variable	Category	Mild (n, %)		Moderate (n, %)		Severe (n, %)		Total (n, %)	
Sex	Female	35	33.02%	6	66.67%	1	25.00%	42	35.29%
	Male	71	66.98%	3	33.33%	3	75.00%	77	64.71%
Education	University (13+ years)	58	54.72%	1	11.11%	0	0.00%	59	49.58%
	High School (9–12 years)	26	24.53%	2	22.22%	1	25.00%	29	24.37%
	Unknown	14	13.21%	3	33.33%	2	50.00%	19	15.97%
	Secondary School (5–8 years)	3	2.83%	3	33.33%	0	0.00%	6	5.04%
	Primary School (1–4 years)	3	2.83%	0	0.00%	0	0.00%	3	2.52%
	No Formal Education	2	1.89%	0	0.00%	1	25.00%	3	2.52%
Employment	Employed	24	22.64%	0	0.00%	2	50.00%	26	21.85%
	Retired	64	60.38%	7	77.78%	0	0.00%	71	59.66%
	Unknown	9	8.49%	1	11.11%	1	25.00%	11	9.24%
	Other	2	1.89%	0	0.00%	0	0.00%	2	1.68%
	Unemployed	7	6.60%	1	11.11%	1	25.00%	9	7.56%
Marital status	Married	49	46.23%	4	44.44%	1	25.00%	54	45.38%
	Single	19	17.92%	2	22.22%	1	25.00%	22	18.49%
	Unknown	14	13.21%	2	22.22%	2	50.00%	18	15.13%
	Living Together	2	1.89%	0	0.00%	0	0.00%	2	1.68%
	Separated	3	2.83%	0	0.00%	0	0.00%	3	2.52%
	Widowed	19	17.92%	1	11.11%	0	0.00%	20	16.81%
Residence	Urban	75	70.75%	9	100.00%	3	75.00%	87	73.11%
	Rural	31	29.25%	0	0.00%	1	25.00%	32	26.89%
Post-traumatic amnesia	No	78	73.58%	6	66.67%	2	50.00%	86	72.27%
	Yes	28	26.42%	3	33.33%	2	50.00%	33	27.73%
Place of injury	Home	69	65.09%	7	77.78%	0	0.00%	76	63.87%
	Work	1	0.94%	0	0.00%	1	25.00%	2	1.68%
	Other	7	6.60%	0	0.00%	0	0.00%	7	5.88%
	Street	28	26.42%	2	22.22%	3	75.00%	33	27.73%
	Sports Facility	1	0.94%	0	0.00%	0	0.00%	1	0.84%
Cause of injury	Fall	80	75.47%	8	88.89%	3	75.00%	91	76.47%
	Other	2	1.89%	0	0.00%	0	0.00%	2	1.68%
	Aggression	3	2.83%	0	0.00%	0	0.00%	3	2.52%
	Road Traffic Accident	21	19.81%	1	11.11%	1	25.00%	23	19.33%
Mechanism injury	Fall From Height > 1 m (3ft)	16	15.09%	2	22.22%	0	0.00%	18	15.13%
	Ground-level Fall	65	61.32%	5	55.56%	2	50.00%	72	60.50%
	Other Non-penetrating TBIs	1	0.94%	0	0.00%	0	0.00%	1	0.84%
	Direct Impact: Blow To the Head	4	3.77%	0	0.00%	0	0.00%	4	3.36%
	Missing Data	3	2.83%	1	11.11%	0	0.00%	4	3.36%
	Acceleration/Deceleration	4	3.77%	1	11.11%	0	0.00%	5	4.20%
	Crush	1	0.94%	0	0.00%	1	25.00%	2	1.68%
	Direct Impact: Head Against an Object	12	11.32%	0	0.00%	1	25.00%	13	10.92%
Type of injury	Closed	104	98.11%	8	88.89%	2	50.00%	114	95.80%
	Penetrating	1	0.94%	0	0.00%	1	25.00%	2	1.68%
	Crush	1	0.94%	1	11.11%	1	25.00%	3	2.52%
Modified Marshall Score	II	91	85.85%	7	77.78%	2	50.00%	100	84.03%
	III	7	6.60%	1	11.11%	1	25.00%	9	7.56%
	I	7	6.60%	1	11.11%	0	0.00%	8	6.72%
	IV	1	0.94%	0	0.00%	0	0.00%	1	0.84%
	V	0	0.00%	0	0.00%	1	25.00%	1	0.84%
Discharge status	Alive	100	94.34%	4	44.44%	3	75.00%	107	89.92%
	Dead	6	5.66%	5	55.56%	1	25.00%	12	10.08%
Discharge to	Home	98	92.45%	4	44.44%	2	50.00%	104	87.39%
	Another Hospital	1	0.94%	0	0.00%	1	25.00%	2	1.68%
	Other	6	5.66%	5	55.56%	1	25.00%	12	10.08%
	Rehabilitation	1	0.94%	0	0.00%	0	0.00%	1	0.84%

Severe (GCS ≤ 8), moderate (GCS 9–12), and mild (GCS 13–15).

## Data Availability

The data presented in this study are available on request from the corresponding author.
